# Is erectile dysfunction associated with osteoarthritis and rheumatoid arthritis? Insights from a population-based study

**DOI:** 10.1093/sexmed/qfae028

**Published:** 2024-06-02

**Authors:** Yuxin Yan, Liyu Zhou, Rui La, Wu Xu, Lisong Li, Dinghua Jiang, Lixin Huang, Qian Wu

**Affiliations:** Department of Orthopedic Surgery, The First Affiliated Hospital of Soochow University, Suzhou, Jiangsu 215000, China; Department of Orthopedic Surgery, The First Affiliated Hospital of Soochow University, Suzhou, Jiangsu 215000, China; Department of Orthopedic Surgery, The First Affiliated Hospital of Soochow University, Suzhou, Jiangsu 215000, China; Department of Orthopedic Surgery, The First Affiliated Hospital of Soochow University, Suzhou, Jiangsu 215000, China; Department of Orthopedic Surgery, The First Affiliated Hospital of Soochow University, Suzhou, Jiangsu 215000, China; Department of Orthopedic Surgery, The First Affiliated Hospital of Soochow University, Suzhou, Jiangsu 215000, China; Department of Orthopedic Surgery, The First Affiliated Hospital of Soochow University, Suzhou, Jiangsu 215000, China; Department of Orthopedic Surgery, The First Affiliated Hospital of Soochow University, Suzhou, Jiangsu 215000, China; Research Institute of Clinical Medicine, Jeonbuk National University Medical School, Jeonju 54896, Republic of Korea

**Keywords:** erectile dysfunction, osteoarthritis, rheumatoid arthritis, National Health and Nutrition Examination Survey, cross-sectional study

## Abstract

**Background:**

The correlation between osteoarthritis (OA) and rheumatoid arthritis (RA), both significant components of arthritis, and erectile dysfunction (ED) has yet to be thoroughly investigated.

**Aim:**

In this study we aimed to assess the association of OA and RA with ED.

**Methods:**

In this observational study we used data from the National Health and Nutrition Examination Survey, which was conducted between 2001 and 2004. Various statistical analyses were employed to investigate the associations of OA and RA with ED, including multivariable logistic regression analysis and subgroup analysis.

**Outcomes:**

The primary outcome for this investigation was arthritis as assessed through self-reporting.

**Results:**

In this comprehensive nationally representative survey spanning 4 years, our findings revealed a notably elevated incidence of ED within both OA and RA populations in comparison to the general population. Additional research is imperative to provide a deeper understanding of these correlations and their potential implications for both pathogenesis and treatment strategies.

**Clinical Implications:**

The research outcomes reported here may serve as a valuable guide for clinicians to assist OA and RA patientsin staying vigilant in addressing their sexual health concerns.

**Strengths and Limitations:**

We explored the association of OA and RA with ED. However, this is only a cross-sectional study.

**Conclusion:**

In this comprehensive nationally representative survey spanning 4 years, our findings revealed a notably elevated incidence of ED within both OA and RA patient populations in comparison to the general population. Ongoing research is imperative to provide a deeper understanding of these correlations.

## Introduction

Arthritis, a group of chronic disorders impacting the joints that affects approximately 25.9% of all adults, stands as a significant facet of global health concerns, manifesting in various forms, with osteoarthritis (OA) and rheumatoid arthritis (RA) being two prominent entities within this category.^1^ The global prevalence of osteoarthritis OA is around 7%, while the prevalence of rheumatoid arthritis (RA) stands at approximately 1%.^2^^,^^3^ The most common form of arthritis, OA, arises from the gradual wear and tear of joint cartilage over time and involves changes in other joint structures like bone and soft tissues, leading to pain, stiffness, and compromised mobility, primarily affecting weight-bearing joints like knees, hips, and spine.^4^ Conversely, RA, an autoimmune disorder, instigates inflammation within the synovial membrane of joints, resulting in joint pain, swelling, and potential deformities.^5^ The prevalence of these conditions is substantial, with OA affecting millions of people globally, particularly among the aging population, and RA affecting individuals across different age groups and occurring more commonly in women.^6^ Both OA and RA affect not only physical function but also overall quality of life, affecting the ability to perform daily activities and potentially causing significant discomfort.^7^^,^^8^ Despite the different etiologies, the burden of pain and functional impairment shared by patients with OA and RA brings these conditions into focus. Understanding the pathophysiological mechanisms, risk factors, and potential associations of OA and RA with other health conditions is of paramount importance as it contributes to the development of effective management strategies, interventions, and public health policies.

Erectile dysfunction (ED), a prevalent and intricate medical condition, is characterized by the recurring inability to achieve or sustain an erection sufficient for satisfactory sexual intercourse.^9^ This disorder encompasses a spectrum of factors that hinder the physiological processes involved in attaining and maintaining an erection. Affecting both the physical and emotional aspects of sexual well-being in men,^10^ ED can arise from various causes, ranging from physiological factors such as cardiovascular disease, diabetes, hormonal imbalances, and neurological disorders, to psychological elements like stress, anxiety, depression, and relationships.^11^^,^^12^ As a condition that transcends mere physical manifestations, ED often carries profound emotional and psychological repercussions, affecting self-esteem, intimate relationships, and overall quality of life.^13^^,^^14^ The prevalence of ED increases with age, but it is by no means restricted to older men, having adverse impacts in a diverse range of individuals across various age groups. An unsettling reality underscores the prevalence of this issue, with 1 of every 2 men older than 40 years grappling with ED.^15^ As medical understanding and risk assessment have continued to evolve, ED has attracted significant attention, and researchers have been exploring the associations of ED with other diseases, such as cardiovascular disease, periodontal disease, sleep disturbance, and systemic sclerosis.^16–18^

The intricate interplay between ED and musculoskeletal disorders presents a fascinating yet relatively uncharted terrain in the realm of medical research. Although the connections between sexual health and musculoskeletal conditions might not appear immediately evident, emerging evidence suggests that there could be underlying links that deserve exploration. According to Deniz et al,^19^ patients with sarcopenia had an almost 30% higher chance of having moderate to severe ED compared to those without (70.8% vs 42.1%，*P* < .001). In a rigorous meta-analysis, Xu et al^20^ meticulously dissected data from a substantial participant pool comprising 22 312 individuals, revealing that patients with ED had a higher risk of osteoporosis than patients without ED (odds ratio [OR], 2.66; 95% CI, 1.42-4.98; *P* = .002). In a separate investigation, Wilton et al^21^ employed a comprehensive population-based cohort approach to delve into potential interplays between various health conditions. Wilton et al’s findings tentatively indicated a connection between men with psoriatic arthritis and men with ED (hazard ratio, 1.45; 95% CI, 0.79-2.68). It is worth noting, however, that this specific relationship did not attain statistical significance within the framework of their study. While the etiology of these conditions might differ significantly from the causes of ED, shared risk factors, biological mechanisms, or even psychological impacts could provide unexpected points of intersection. Given that OA and RA are among the most prevalent conditions affecting the bone and joint system, our curiosity was piqued by the possibility of a potential correlation with ED.

Hence, the objective of this study was to investigate the potential association between ED and OA and RA through an encompassing cross-sectional analysis, leveraging the expansive dataset provided by the National Health and Nutrition Examination Survey (NHANES).

## Methods

### Study population in NHANES

The NHANES study stands as a comprehensive cross-sectional survey conducted within the United States. Through employment of a meticulously structured stratified multistage random sampling methodology,^22^ the NHANES primary objective is to gather extensive data about the overall health and nutritional well-being of the general populace. Ethical validation for NHANES was granted by the National Center for Health Statistics study ethical review board, and each participant provided a signed informed consent form. The NHANES datasets, accompanied by thorough documentation and protocols, are openly accessible for retrieval from the designated website.

**Figure 1:**
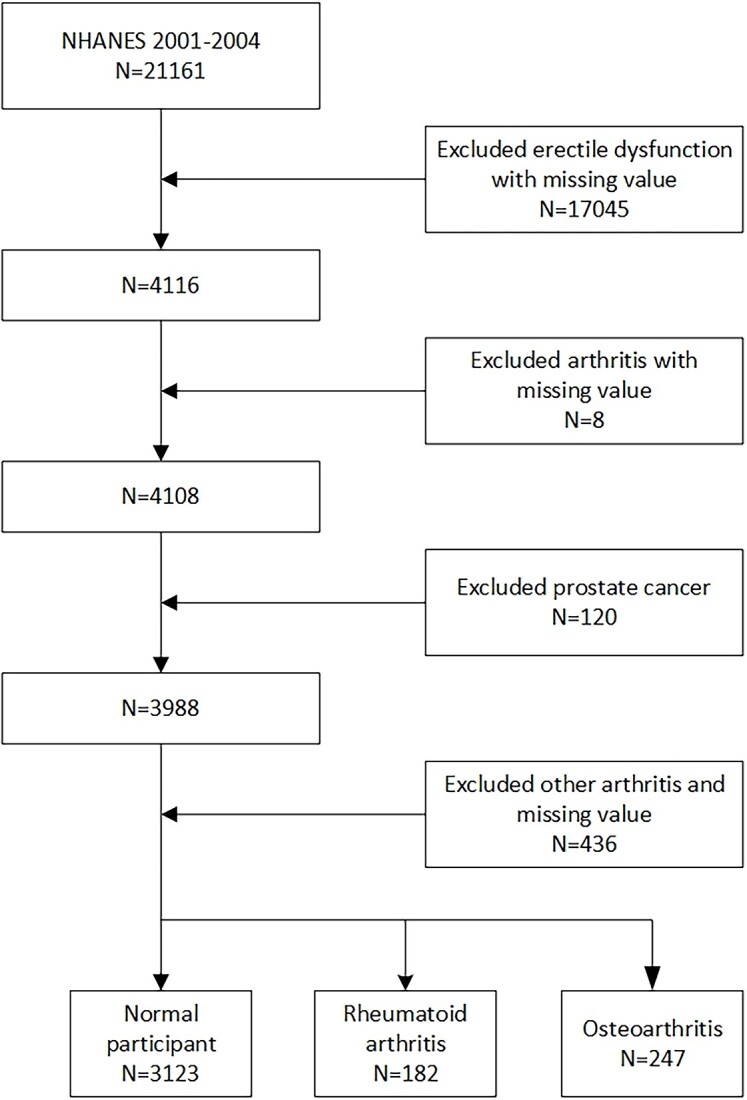
Flowchart of the participant selection.

In this study, we utilized data from the NHANES 2001-2004 cycles owing to their incorporation of information about both ED and arthritis. Rigorous measures were taken to ensure the accuracy and dependability of our findings. Our investigation involved the application of specific exclusion criteria, meticulously chosen to bolster the credibility of our analysis. Exclusion criteria were as follows: (1) Individuals with incomplete ED data, to maintain data integrity. (2) Individuals with incomplete arthritis data, as their inclusion might introduce potential biases into our analytical framework. (3) Individuals with a history of prostate cancer, as this could confound the relationship under study. (4) Individuals with RA and other arthritis were excluded to focus on participants with OA, or individuals with OA and other arthritis were excluded to focus on participants with RA. For a more in-depth comprehension of our study design, the methods of sampling employed, and the specific criteria for exclusions, we direct interested readers to Figure 1.

### Assessment of ED

The definition of ED in this study was based on participant responses to a survey question that has been validated to reliably identify men clinically diagnosed with ED.^23^ The question posed was as follows: “Many men experience problems with sexual intercourse. How would you describe your ability to get and keep an erection adequate for satisfactory intercourse?” Response options included “always or almost always able,” “usually able,” “sometimes able,” and “never able.” In this analysis, the responses “sometimes able” or “never able” to keep an erection were classified as having ED and “always or almost always able” or “usually able” to keep an erection were classified as not having ED.

### Assessment of OA and RA

According to our previous research reports,^24–26^ the data concerning arthritis diagnosis was derived from self-reported information obtained during personal interviews with participants. The survey inquired whether a healthcare professional had ever communicated a diagnosis of arthritis to the participant. If the response was affirmative, a subsequent query followed: “Which type of arthritis was it?” This enabled the classification of arthritis into distinct categories such as OA and RA.

### Assessment of covariables of interest

To mitigate the potential influences of confounding factors, adjustments were made for a range of demographic characteristics, ensuring the integrity of our analysis. These included age, race, level of education, poverty-to-income ratio (PIR), marital status, body mass index (BMI), C-reactive protein (CRP), hypertension, diabetes, smoking status, vigorous activity and moderate activity. Age was categorized into two groups: “<65 and ≥65 years” enabling comparison of the effects between participants of different age ranges. Marital status was divided into two distinct categories: “Married or with a partner” and “Single,” allowing the discernment of potential disparities linked to relationship status. The smoking habits of participants were delineated into three classifications: “Never smokers,” “Former smokers,” and “Current smokers.” This differentiation was contingent on whether participants had smoked fewer than 100 cigarettes over their lifetime and their present smoking behavior. The latex-enhanced nephelometry method was used to quantify CRP, which is considered one of the best measures of tissue damage and inflammation. Details of CRP measurement process and other covariate acquisition process are available at www.cdc.gov/nchs/nhanes/.

### Statistical analysis

Within the framework of this observational investigation utilizing NHANES data, we employed multivariate logistic regression analysis to delve into the intricate interplay of the association of OA and RA with ED. Demographic characteristics and prevalence of covariables of the OA and RA cohorts separately by means of baseline characteristics table. Categorical variables were compared with the Chi-square test. Our analytical approach involved three distinct models to progressively refine our understanding. In model 1, no adjustments were applied, allowing for an initial assessment of the unadulterated relationship. Model 2 accounted for the influences of age and race, extending the analysis to a more nuanced level. Ultimately, model 3 emerged as the central focus, encompassing the variables of model 2 as well as the level of education, PIR, marital status, BMI, CRP, hypertension, diabetes, smoking status, vigorous activity, and moderate activity. Continuous variables were presented as mean (SD), whereas categorical variables were expressed as frequencies and percentages.

All statistical analyses were meticulously executed through the utilization of R software (version 4.1.3) and EmpowerStats (version 2.0). The threshold for statistical significance was established at *P* < .05, encapsulating the level at which findings were deemed notable within the scope of our exploration.

## Results

### Baseline characteristics of participants with OA and the general population

Table 1 outlines the baseline characteristics of the OA group and the general population within the study. A total of 3370 participants fulfilled the selection criteria and were included, resulting in an overall OA incidence of 7.33% within this cohort. Among these participants, 78.81% were under the age of 65 years, while 21.19% were 65 years or older. Marital status distribution revealed that 66.89% were categorized as “Married or with a partner,” with the remaining 33.11% identified as “Single.” Regarding ethnicity, 21.54% were of Mexican American descent, 3.83% were from other Hispanic backgrounds, 52.23% were non-Hispanic Whites, 19.23% were non-Hispanic Blacks, and 3.18% belonged to other racial groups. The mean (SD) concentration ranges of the PIR, BMI, and CRP values were 2.83 (1.61), 27.81 (5.46) kg/m^2^ and 0.35 (0.83) mg/dL, respectively.

**Table 1:** Baseline characteristics of participants with OA and the general population.

	**Overall**	**ED**	**Non-ED**	***P* value**
Age in years, %				<.001
<65	78.81	42.58	90.37	
≥65	21.19	57.42	9.63	
Race, %				.001
Mexican American	21.54	22.58	21.21	
Other Hispanic	3.83	4.17	3.72	
Non-Hispanic White	52.23	56.20	50.96	
Non-Hispanic Black	19.23	14.72	20.67	
Other races	3.18	2.33	3.44	
Marital status, %				<.001
Married or with partner	66.89	72.27	65.18	
Single	33.11	27.73	34.82	
Education level, %				<.001
Less than high school	26.69	39.51	22.60	
High school or GED	24.82	19.63	26.48	
Above high school	48.49	40.86	50.92	
Smoking status, %				<.001
Never	42.34	30.14	46.18	
Former	29.82	48.83	23.80	
Current	27.83	21.03	30.02	
Diabetes, %				<.001
Yes	8.72	21.53	4.67	
No	91.28	78.47	95.33	
Hypertension, %				<.001
Yes	27.38	48.88	20.53	
No	72.62	51.12	79.47	
Vigorous activity, %				<.001
Yes	37.55	18.99	43.08	
No	62.45	81.01	56.92	
Moderate activity, %				<.001
Yes	50.60	47.48	52.52	
No	49.40	55.67	47.48	
PIR, mean (SD)	2.83 (1.61)	2.54 (1.55)	2.92 (1.62)	<.001
BMI, mean (SD)	27.81 (5.46)	28.17 (5.84)	27.70 (5.33)	.090
CRP, mean (SD)	0.35 (0.83)	0.45 (0.87)	0.32 (0.81)	<.001
OA, %				<.001
Yes	7.33	16.56	4.38	
No	92.67	83.44	95.62	

Abbreviations: BMI, body mass index; CRP, C-reactive protein; ED, erectile dysfunction; OA, osteoarthritis; PIR, poverty-to-income ratio; RA, rheumatoid arthritis.

Comparison between participants with and without ED revealed a lower prevalence of being younger than 65 years of age, being single, having a current smoking status, engaging in vigorous activity, and participating in moderate activity among individuals with ED (all *P* < .01). Upon further analysis, the prevalence of OA was observed to be higher among participants with ED in contrast to those without (non-ED participants).

### Association between ED and OA

Table 2 presents the outcomes of our multivariate logistic regression analysis investigating the connection between ED and OA. The initial unadjusted model unveiled a markedly substantial and positive correlation between ED and OA (OR, 4.33; 95% CI, 3.32-5.64; *P* < .0001). When introducing gender, age, and race as variables in model 2, this notable positive correlation persisted (OR, 2.60; 95% CI, 1.88-3.60; *P* < .0001). Remarkably, even with the comprehensive inclusion of all covariates in model 3, the noteworthy association between ED and OA remained both significant and positive (OR, 2.13; 95% CI, 1.47-3.09; *P* < .0001).

**Table 2:** Association and subgroup analyses between ED and OA.^a^

	**OR (95% CI), *P* value**		
	**Model 1 = 3370**	**Model 2 = 3370**	**Model 3 = 2868**
Overall			
No ED	Reference	Reference	Reference
ED	4.33 (3.32-5.64) <.0001	2.60 (1.88-3.60) <.0001	2.13 (1.47-3.09) <.0001
Age in years			
<65	3.88 (2.60-5.80) <.0001	4.48 (2.97-6.75) <.0001	2.88 (1.74-4.77) <.0001
≥65	1.44 (0.95-2.18) .0865	1.42 (0.93-2.17) .1049	1.39 (0.84-2.31) .2030
Marital status			
Married or with partner	4.06 (3.02-5.45) <.0001	2.53 (1.78-3.61) <.0001	2.36 (1.58-3.54) <.0001
Single	4.68 (2.54-8.63) <.0001	2.88 (1.29-6.43) .0096	1.15 (0.42-3.15) .7863
Education level			
Less than high school	2.56 (1.53-4.31) .0004	1.54 (0.84-2.85) .1643	1.49 (0.70-3.16) .3007
High school or GED	7.50 (4.17-13.46) <.0001	4.38 (2.14-8.95) <.0001	4.32 (1.91-9.78) .0005
Above high school	4.96 (3.44-7.15) <.0001	2.70 (1.72-4.24) <.0001	1.85 (1.11-3.10) .0190
Smoking status			
Never	4.08 (2.53-6.57) <.0001	1.83 (1.01-3.33) .0471	1.82 (0.91-3.62) .0892
Former	3.28 (2.23-4.82) <.0001	2.19 (1.39-3.45) .0007	2.12 (1.26-3.58) .0047
Current	3.81 (2.00-7.22) <.0001	4.71 (2.33-9.55) <.0001	3.34 (1.44-7.78) .0050
Diabetes			
Yes	1.44 (0.72-2.88) .2958	1.26 (0.60-2.65) .5468	1.11 (0.46-2.65) .8190
No	5.01 (3.73-6.73) <.0001	2.82 (1.95-4.07) <.0001	2.28 (1.51-3.43) <.0001
Hypertension			
Yes	2.14 (1.48-3.10) <.0001	1.74 (1.14-2.65) .0104	1.64 (1.00-2.67) .0484
No	2.14 (1.48-3.10) <.0001	2.88 (1.77-4.70) <.0001	2.82 (1.62-4.92) .0003
Vigorous activity			
Yes	3.74 (1.98-7.08) <.0001	2.21 (0.99-4.93) .0539	2.04 (0.76-5.43) .1548
No	3.84 (2.80-5.26) <.0001	2.53 (1.74-3.68) <.0001	2.15 (1.43-3.23) .0002
Moderate activity			
Yes	4.38 (3.04-6.33) <.0001	2.80 (1.79-4.40) <.0001	2.34 (1.44-3.79) .0006
No	4.02 (2.66-6.08) <.0001	2.18 (1.31-3.63) .0026	1.77 (0.98-3.20) .0585

Abbreviations: BMI, body mass index; CRP, C-reactive protein; ED, erectile dysfunction; OA, osteoarthritis; OR, odds ratio; PIR, poverty-to-income ratio; RA, rheumatoid arthritis.

a Associations (OR, 95% CI) of ED with RA overall. Model 1: no covariates were adjusted; model 2, adjusted for age and race; model 3: adjusted for age, race, level of education, PIR, marital status, BMI, CRP, hypertension, diabetes, smoking status, vigorous activity, and moderate activity.

Within our subgroup shown in Table 2, a significant association emerged within both participants with and without hypertension within the OA cohort (OR, 1.64; 95% CI, 1.00-2.67, and OR, 2.82; 95% CI, 1.62-4.92, respectively). Specifically, within the subgroup encompassing individuals below 65 years of age, those married or with a partner, those with an educational background above high school, former and current smokers, individuals without diabetes, those engaging in no vigorous activity, and those involved in moderate activity, a substantial link between ED and OA was consistently observed.

### Baseline characteristics of participants with RA and the general population

Table 3 showcases a comprehensive overview of the baseline characteristics observed within the RA group and the general population under investigation. The selected cohort for analysis encompassed a total of 3305 participants, reflecting a cumulative incidence of RA within this collective at 5.51%. Among these individuals, 80.06% were younger than 65 years, while 19.94% were 65 years or older. An examination of marital status distribution revealed that 65.82% were either married or in a committed partnership, with the remaining 34.18% identified as single. Regarding ethnicity, 22.15% were of Mexican American descent, 3.93% were from other Hispanic backgrounds, 50.62% were non-Hispanic Whites, 20.03% were non-Hispanic Blacks, and 3.27% belonged to other racial groups. The baseline metrics further encompassed pivotal parameters: the mean (SD) concentrations for PIR, BMI, and CRP values were 2.79 (1.62) and 27.72 (5.46) kg/m^2^, and 0.37 (0.89) mg/dL, respectively. Notably, a significant distinction emerged in the prevalence of several factors when comparing participants with ED and those without. Specifically, the prevalence of characteristics such as age below 65 years, single marital status, nonsmoking status, absence of diabetes, absence of hypertension, engagement in vigorous activity, and engagement in moderate activity was notably lower among participants experiencing ED as opposed to those without this condition (all *P* < .01). Upon further stratification, it was evident that the prevalence of RA was notably higher among participants experiencing ED, in comparison to those without ED.

**Table 3:** Baseline characteristics of participants with RA and the general population.

	**Overall**	**ED**	**Non-ED**	***P* value**
Age in years, %				<.001
<65	80.06	43.79	90.98	
≥65	19.94	56.21	9.02	
Race, %				.033
Mexican American	22.15	24.44	21.46	
Other Hispanic	3.93	4.44	3.78	
Non-Hispanic White	50.62	51.90	50.24	
Non-Hispanic Black	20.03	16.60	21.06	
Other races	3.27	2.61	3.46	
Marital status, %				.003
Married or with partner	65.82	70.33	64.46	
Single	34.18	29.67	35.54	
Education level, %				<.001
Less than high school	27.49	42.48	22.97	
High school or GED	25.19	19.74	26.83	
Above high school	47.32	37.78	50.20	
Smoking status, %				<.001
Never	41.94	30.46	45.39	
Former	28.90	46.41	23.62	
Current	29.17	23.14	30.98	
Diabetes, %				<.001
Yes	8.58	22.56	4.42	
No	91.42	77.44	95.58	
Hypertension, %				<.001
Yes	26.52	48.34	19.97	
No	73.48	51.66	80.03	
Vigorous activity, %				<.001
Yes	37.69	19.83	42.72	
No	62.31	80.17	57.28	
Moderate activity, %				<.001
Yes	49.83	42.10	52.08	
No	50.17	57.90	47.92	
PIR, mean (SD)	2.79 (1.62)	2.46 (1.54)	2.89 (1.63)	<.001
BMI, mean (SD)	27.72 (5.46)	27.96 (5.67)	27.65 (5.40)	.307
CRP, mean (SD)	0.37 (0.89)	0.33 (0.82)	0.51 (1.10)	<.001
RA, %				<.001
Yes	5.51	11.11	3.82	
No	94.49	88.89	96.18	

Abbreviations: BMI, body mass index; CRP, C-reactive protein; ED, erectile dysfunction; PIR, poverty-to-income ratio; RA, rheumatoid arthritis.

### Association between ED and RA

Table 4 presents the outcomes derived from a comprehensive multivariate logistic regression analysis investigating the interplay between ED and RA. The initial unadjusted model revealed a notably robust and statistically significant positive correlation between ED and RA (OR, 3.15; 95% CI, 2.32-4.26; *P* < 0.0001). Progressing to model 2, wherein adjustments were introduced for essential factors encompassing gender, age, and race, the profound positive association persisted with marked significance (OR, 2.22; 95% CI, 1.54-3.20; *P* < 0.0001). Furthermore, upon meticulous incorporation of all pertinent covariates in model 3, the robust link between ED and RA continued to hold prominence (OR, 1.67; 95% CI, 1.08-2.57; *P* = 0.0216).

**Table 4:** Association and subgroup analysis between ED and RA.^a^

	**OR (95% CI), *P* value**		
	**Model 1 = 3305**	**Model 2 = 3305**	**Model 3 = 2814**
Overall			
No ED	Reference	Reference	Reference
ED	3.15 (2.32-4.26) <.0001	2.22 (1.54-3.20) <.0001	1.67 (1.08-2.57) .0216
Age, years			
<65	2.65 (1.69-4.14) <.0001	2.93 (1.86-4.62) <.0001	2.14 (1.24-3.71) .0065
≥65	1.60 (0.93-2.73) .0874	1.63 (0.95-2.79) .0772	1.23 (0.64-2.34) .5393
Marital status			
Married or with a partner	3.19 (2.21-4.61) <.0001	2.44 (1.58-3.77) <.0001	1.68 (1.00-2.83) .0487
Single	3.02 (1.75-5.20) <.0001	1.65 (0.82-3.32) .1567	1.69 (0.75-3.83) .2075
Education level			
Less than high school	2.27 (1.40-3.69) .0009	1.65 (0.94-2.90) .0803	1.75 (0.89-3.44) .1049
High school or GED	4.30 (2.36-7.82) <.0001	2.54 (1.20-5.38) .0145	1.85 (0.73-4.67) .1923
Above high school	2.95 (1.74-4.99) <.0001	2.31 (1.21-4.41) .0108	1.50 (0.68-3.29) .3144
Smoking status			
Never	6.31 (3.19-12.46) <.0001	3.27 (1.41-7.62) .0059	2.72 (0.99-7.48) .0528
Former	1.98 (1.25-3.14) .0035	1.49 (0.86-2.56) .1513	1.14 (0.58-2.24) .6976
Current	2.71 (1.60-4.61) .0002	2.33 (1.29-4.22) .0053	2.21 (1.10-4.41) .0250
Diabetes			
Yes	2.58 (1.07-6.19) .0338	2.23 (0.89-5.59) .0886	3.83 (1.06-13.82) .0398
No	2.96 (2.09-4.18) <.0001	1.97 (1.29-3.01) .0016	1.45 (0.89-2.34) .1329
Hypertension			
Yes	1.97 (1.27-3.06) .0025	1.91 (1.16-3.14) .0108	1.97 (1.06-3.66) .0332
No	3.01 (1.93-4.70) <.0001	1.76 (1.02-3.02) .0420	1.48 (0.79-2.77) .2166
Vigorous activity			
Yes	5.81 (2.71-12.45) <.0001	4.37 (1.77-10.79) .0014	2.20 (0.73-6.65) .1607
No	2.21 (1.55-3.16) <.0001	1.70 (1.12-2.58) .0132	1.51 (0.94-2.43) .0870
Moderate activity			
Yes	1.51 (0.94-2.43) .0870	2.44 (1.35-4.40) .0030	1.64 (0.85-3.17) .1386
No	3.22 (2.12-4.88) <.0001	2.03 (1.23-3.34) .0055	1.70 (0.94-3.07) .0806

Abbreviations: BMI, body mass index; CRP, C-reactive protein; ED, erectile dysfunction; OR, odds ratio; PIR, poverty-to-income ratio; RA, rheumatoid arthritis.

a Associations (OR, 95% CI) of ED with RA overall. Model 1: no covariates were adjusted; model 2, adjusted for age, and race; model 3: adjusted for age, race, level of education, PIR, marital status, BMI, CRP, hypertension, diabetes, smoking status, vigorous activity, and moderate activity.

In a subgroup focusing on individuals below the age of 65 years, those who were married or had a partner, those who were current smokers, and those with a history of diabetes and hypertension, a significant connection emerged between ED and RA.

## Discussion

The results of this population-based study make a substantial contribution to our comprehension of the interrelationship between ED and two prevalent musculoskeletal disorders, OA and RA. Our findings underscore a prominent positive correlation between these seemingly disparate conditions, persisting even after meticulous adjustments for an array of covariates.

While OA continues to grow as a prevalent health concern, a gap persists in our understanding of how various individual risk factors intertwine to impact its development and progression.^27^ A number of common risk factors, including obesity, joint injuries, subluxations and deformities, low muscle strength, and limb inequality, have been studied extensively. However, a novel line of research, the link between ED and OA, seems to have caught the attention of researchers. To begin with, both OA and ED are associated with ageing. The former is most common in middle-aged and older adults, while the latter mainly affects men over the age of 40 years.^28^ Suffering from ED is a signal of physical deterioration, which naturally includes bones and joints. Previous studies have also considered the impact of OA on sexual function. Not surprisingly, patients with OA suffer from pain and limited joint mobility, affecting sexual positional freedom and leading to reduced sexual satisfaction.^29^ Nevertheless, when OA patients underwent joint replacement surgery to achieve satisfactory joint mobility, their sexual performance improved.^30^ It must be recognized that the poor quality and low frequency of sex in OA patients is both physically and psychologically devastating, which undoubtedly affects erectile function in men. In turn, patients with poor erectile function often need to change sexual positions in order to achieve greater sexual satisfaction, and prolonged inappropriate positions will inevitably increase joint wear and tear, thereby increasing the risk of OA. In addition, patients with ED often exhibit the following characteristics: obesity, physical inactivity, unhealthy diet, dyslipidemia, and neurological and psychiatric problems, which may also represent risk factors for OA.^31^^,^^32^ Regrettably, there are fewer studies investigating the interaction between ED and OA. Although Blake et al^33^ demonstrated an increased likelihood of ED among arthritis patients compared to the general population, their study did not delve into the nuanced differences between RA, OA, and spondyloarthropathy due to sample constraints. Research investigating whether ED serves as a potential precursor to OA remains scarce. Notably, this study marks the inaugural endeavor of its kind—leveraging a substantial population-based dataset—to examine the intricate relationship between ED and OA. Our findings reveal a remarkable ED prevalence of 16.56% among OA-diagnosed individuals, significantly surpassing the rates of 4.38% among non-OA counterparts and 7.33% within the NHANES cohort. This result tentatively suggests a possible link between ED and OA. The intricate association between these two conditions is likely influenced by underlying mechanisms that warrant further exploration. While the exact nature of this relationship is yet to be fully unraveled, shared pathophysiological factors may play a pivotal role. Both conditions exhibit notable components of vascular dysfunction, inflammation, and hormonal imbalances. The systemic inflammation inherent in OA could potentially contribute to endothelial dysfunction, affecting blood flow to the erectile tissues and manifesting as ED.^34^^,^^35^ Furthermore, common risk factors like obesity, sedentary lifestyle, and metabolic syndrome could contribute synergistically to the development of both OA and ED.^36^^,^^37^ Hormonal imbalances, particularly those affecting the role of testosterone in maintaining both musculoskeletal and erectile function, could also serve as a link between the two conditions.^38^^,^^39^

Then the attention turns to RA and ED, which may show a closer connection. As a systemic immune system disease, RA attacks not only the bones, joints, and soft tissues in patients, but also targets cardiovascular, respiratory, neurological, and other systems, resulting in an intractable comorbidity.^40^ The reproductive health of people with RA, especially sexual health, is also an issue that deserves attention, as it plays an increasingly important role in an individual's quality of life and level of physical and mental health. On the one hand, the complexity of RA has a negative impact on sexual health; RA patients also suffer from fatigue, chronic pain, limited joint mobility, and even disability, and these physical distresses are accompanied by psychological uncertainty, which may lead to a lower quality of sexual life for RA patients, which manifests itself as ED in men.^41^ On the other hand, unsatisfactory sexual life will lead to emotional disorders such as depression and anxiety, and even sociological problems such as poor relationships between couples and breakdown of marital status, which would be another psychological shock for RA patients and may aggravate the condition of RA. Moreover, sexual dysfunction is associated with health factors such as physical activity, weight, and smoking, which bridge the gap with RA.^42^ As a result, the relationship between RA and sexual dysfunction has become brighter and brighter, attracting more and more researchers to look for the exact relationship between them.

In a comprehensive exploration of the relationship between sexual dysfunction and RA, a series of studies have provided compelling insights into the multifaceted nature of this association. Keller et al^43^ highlighted that diminished functional capacity in RA patients corresponded to reduced sexual motivation, with a substantial proportion seeking expert advice for sexual problems. Other investigations corroborate these findings, demonstrating a higher prevalence of sexual dysfunction in RA patients compared to controls. Notably, impotence emerged as a significant concern among RA patients, further emphasizing the complexity of the observed association.^33^ The multifactorial nature of sexual dysfunction in RA was underscored by an Egyptian multicenter study, which revealed correlations between sexual dysfunction and factors intrinsic to RA, including pain, cardiovascular disease, disease activity, fatigue, and psychological status.^41^ Similarly, in line with our findings, the prevalence of ED among patients with RA was notably higher at 11.11%, markedly surpassing the rates of 3.82% observed among non-RA participants and the broader general population prevalence of 5.51%. The intricate relationship between RA and ED is characterized by complex underlying mechanisms that warrant in-depth investigation. While the precise mechanisms that link these two conditions are yet to be fully investigated, there are intriguing connections that invite exploration. Both RA and ED share common pathways involving inflammation and immune dysregulation. The chronic inflammatory state seen in RA may impact endothelial function and vascular health, thereby contributing to the development of ED.^44–47^ Moreover, the psychological and emotional burden associated with chronic pain and disability in RA could potentially play a role in the onset or exacerbation of ED.^48–51^ Shared risk factors such as smoking, obesity, and a sedentary lifestyle could further contribute to the observed association.^52^^,^^53^

From a clinical perspective, recognizing these associations could lead to improved patient care. Healthcare practitioners managing patients with OA or RA should be vigilant in assessing sexual health concerns, considering the potential impact of ED on the overall well-being of these individuals. Conversely, urologists and sexual health specialists should be attuned to the heightened prevalence of OA and RA among their patients, potentially prompting early screening and intervention for musculoskeletal issues.

While our study establishes a noteworthy positive association between ED, OA, and RA, it is crucial to acknowledge some limitations. The cross-sectional nature of our analysis restricts our ability to infer causality. Longitudinal studies are warranted to unravel the temporal aspects of this relationship and investigate whether ED contributes to OA and RA or vice versa. Moreover, the reliance on self-reported data introduces the potential for recall bias. Furthermore, while this investigation incorporated a multitude of covariates aiming to attenuate the impact of confounding variables, it nonetheless overlooked critical potential determinants influencing the nexus between ED, OA, and RA. Notably, the analysis did not encompass considerations pertinent to prostate cancer, severe cardiovascular incidents, pharmacological interventions, and sociopsychological determinants. Moreover, while our findings provide valuable insights into the correlation between ED, OA, and RA, the precise mechanisms underlying this relationship remain speculative. It is essential to consider the complex interplay of physical, psychological, and biological factors that could contribute to this observed association.

## Conclusion

In conclusion, our study provides compelling evidence for a substantial and consistent positive correlation between ED, OA, and RA. This study emphasizes the importance of interdisciplinary collaboration among urologists, rheumatologists, and other specialists to comprehensively address the interconnected health concerns of patients with ED, OA, and RA.

## Data Availability

All data applied in this study can be searched on the NHANES website (https://wwwn.cdc.gov/nchs/nhanes/Default.aspx), and more detailed analysis data can be obtained by contacting the corresponding author.
